# A Realistic Approach for Photoelectrochemical Hydrogen Production

**DOI:** 10.3390/ma11081269

**Published:** 2018-07-24

**Authors:** Elias Doukas, Paraskevi Balta, Dimitrios Raptis, George Avgouropoulos, Panagiotis Lianos

**Affiliations:** 1 Department of Materials Science, University of Patras, 26500 Patras, Greece; eldouk1@gmail.com (E.D.); geoavg@upatras.gr (G.A.); 2 Department of Chemical Engineering, University of Patras, 26500 Patras, Greece; vivibalta@windowslive.com (P.B.); dgraptis86@yahoo.gr (D.R.)

**Keywords:** hydrogen, photoelectrocatalytic, photocatalytic fuel cell, carbon electrode

## Abstract

The production of hydrogen by water splitting has been a very attractive idea for several decades. However, the energy consumption that is necessary for water oxidation is too high for practical applications. On the contrary, the oxidation of organics is a much easier and less energy-demanding process. In addition, it may be used to consume organic wastes with a double environmental benefit: renewable energy production with environmental remediation. The oxidation of organics in a photoelectrochemical cell, which in that case is also referenced as a photocatalytic fuel cell, has the additional advantage of providing an alternative route for solar energy conversion. With this in mind, the present work describes a realistic choice of materials for the Pt-free photoelectrochemical production of hydrogen, by employing ethanol as a model organic fuel. The photoanode was made of a combination of titania with cadmium sulfide as the photosensitizer in order to enhance visible light absorbance. The cathode electrode was a simple carbon paper. Thus, it is shown that substantial hydrogen can be produced without electrocatalysts by simply exploiting carbon electrodes. Even though an ion transfer membrane was used in order to allow for an oxygen-free cathode environment, the electrolyte was the same in both the anode and cathode compartments. An alkaline electrolyte has been used to allow high hydroxyl concentration, thus facilitating organic fuel (photocatalytic) oxidation. Hydrogen production was then obtained by water reduction at the cathode (counter) electrode.

## 1. Introduction

Hydrogen production by water splitting has been one of the most attractive research subjects for several decades. Hydrogen offers the highest gravimetric heat of combustion, ~286 kJ/mole, and is the most benign of all of the fuels, since its combustion simply reproduces water. However, water splitting is a rather high energy-demanding process. In the case of water electrolysis, for example, a high overpotential is necessary for water oxidation. Theoretically, water is electrochemically split (oxidized) at +1.23 V, but in reality, much higher voltages are necessary due to losses [1]. In this respect, expensive catalysts are necessary to limit the applied electric potential [2]. A better situation presents itself when in the place of water, an organic agent is electrochemically oxidized, since the oxidation of some organic materials is achieved at lower potentials [3,4]. Seen from a different point of view, the most crucial issue is related to the oxidation of water being a four-electron process:

2H_2_O→O_2_ +4H^+^+4e^−^ (1)

In other words, it is hard to simultaneously extract four units of charge in order to split water and produce molecular oxygen. On the contrary, it is easier to oxidize an organic substance. For example, in the case of ethanol, oxidation leads to the formation of acetaldehyde, which is a two-electron process, and it is more easily realized [5]:

C_2_H_6_O→CH_3_CHO + 2H^+^ + 2e^−^ (2)

It is even easier in an alkaline environment where oxidation is facilitated by the intermediate of hydroxyl radicals [5]:

OH^−^→•OH + e^−^ and C_2_H_6_O + 2•OH→CH_3_CHO + 2H_2_O (3)

Obviously, it is easier to oxidize an organic substance than to oxidize water. Molecular hydrogen production is also a two-electron process, which may be realized either by proton or water reduction [5]:

2H^+^ + 2e^−^→H_2_    (acidic environment) (4)

or 2H_2_O + 2e^−^→2OH^−^ + H_2_   (alkaline environment) (5)

Photocatalytic oxidation is obtained in the presence of a photocatalyst, which absorbs photons producing electron-hole pairs. Oxidation is achieved by electron injection from the substance to be oxidized to the excited semiconductor, thus neutralizing the photogenerated holes. In a photoelectrochemical system, photogenerated electrons are picked by the anode electrode and carried through an external circuit to the cathode electrode; there, they assist reduction reactions, such as for example, hydrogen production under anaerobic conditions. Oxidation then takes place at the (photo) anode electrode, and hydrogen is produced at the cathode electrode. The driving force that leads electrons from the anode to the cathode electrode is the electrochemical potential difference that develops between the two electrodes [6]. Thus, under favorable conditions, the anode potential is more negative than the cathode potential. However, hydrogen production potential is only a bit more positive or even more negative than the conduction band of most known photocatalysts [6]. For this reason, there is no sufficient bias to allow unassisted hydrogen production, which is realized only under externally applied electric bias. Indeed, there exist a lot of data on this matter, and it is well known that an electric bias is always necessary in order to produce hydrogen by photoelectrochemical procedures [7,8,9]. Alternatively, a chemical bias may be applied, for example, by using an alkaline electrolyte at the anode, and an acidic electrolyte at the cathode [10], but this is not convenient for practical applications.

In the present work, hydrogen has indeed been produced by a simple photoelectrochemical reactor using well-known materials and simplified procedures. We have employed nanoparticulate titania as the basic photocatalyst, and combined it with nanoparticulate cadmium sulfide (CdS) as visible light sensitizer. As a counter electrode, we have used either carbon paper alone or decorated with reduced graphene oxide (RGO) or carbon black. Carbon black was also in some cases enhanced with deposited Pt nanoparticles for reasons of comparison. As a sacrificial agent, that is, as fuel to run the cell, we have used ethanol, which was chosen as a model biomass-derived fuel, while an alkaline (NaOH) electrolyte filled the reactor. The above combination of materials is somehow unusual. TiO_2_/CdS photoanodes have mostly been used with sulfide electrolytes [11,12,13]. Nevertheless, the stability of CdS is guaranteed in the presence of an efficient hole scavenger, and this has been repeatedly shown in the presence of ethanol [14,15,16,17]. Any realistic approach for photoelectrocatalytic hydrogen production cannot be envisaged but with the employment of noble metal-free installations. The motivation then of this work was to show that hydrogen can indeed be produced by using a simple photoelectrocatalytic installation and low cost materials, and thus open up routes for cost-effective hydrogen production.

## 2. Materials and Methods

### 2.1. Materials

Unless otherwise specified, reagents were obtained from Sigma Aldrich and were used as received. Millipore water was used in all experiments. SnO_2_:F transparent conductive electrodes (FTO, resistance 8 ohm/square) were purchased from Pilkington and carbon paper was purchased from SGL Technologies GmbH (Meitingen, Germany), (thickness: 190 μm).

### 2.2. Preparation of Photoanode Electrode

CdS/TiO_2_/FTO photoanode electrodes were constructed by following protocols established by previous publications [18,19]. Briefly, nanoparticulate titania films were deposited on FTO transparent electrodes by the following procedure. A FTO glass was cut in the appropriate dimensions, and was carefully cleaned first with soap, and then by sonication in isopropanol, water, and acetone. A thin layer of compact titania was first sprayed over a patterned area by using 0.2 mol L^−1^ diisopropoxytitanium bis (acetylacetonate) solution in ethanol, and was calcined at 500 °C. The deposition of this bottom compact layer is a common practice with nanocrystalline titania photoanodes, since it enhances attachment of the top thick film, prevents short circuits, and facilitates electron flow toward the electrode. On the top of this compact film, we applied a titania paste made of P25 nanoparticles by doctor blading. The film was calcined up to 550 °C at a rate of 20 °C/min. The final thickness of the film, as measured by SEM, was approximately 10 µm. The active geometrical area of the film was 1 cm^2^ (1 cm × 1 cm) in the cases where current–voltage curves were plotted but increased to 5 cm × 3.5 cm for hydrogen production measurements.

CdS was deposited on TiO_2_ films by 10 SILAR (successive ionic layer adsorption and reaction) cycles [18,20] using Cd(NO_3_)_2_ as Cd^2+^ and Na_2_S as S^2−^ precursor. We used 0.1 mol L^−1^ of aqueous solutions for both cations and anions. After SILAR deposition and final washing with three-dimensional (3D) water, the films were dried in an oven at 100 °C and were ready for use.

### 2.3. Construction of the Counter Electrode

Carbon paper (CP) sheets were cut in pieces of 6 cm × 3.5 cm. A band of 1 cm × 3.5 cm was left clear to make electric contact, and the rest (5 cm × 3.5 cm) was either left intact or was covered with an electrocatalyst. For this purpose, we prepared the following pastes based on carbonaceous materials. Graphene oxide (GO) was obtained according to the classical Staudenmaier method [21]. Graphite (2 g) was added to 150 mL of a cold mixture of H_2_SO_4_ and HNO_3_ (2:1) in an ice-water bath. KClO_3_ powder (50 g) was then added in small portions under continuous stirring and cooling. After 20 h, the reaction was quenched by pouring the mixture into distilled water. GO was isolated by filtration and purified extensively with water (until the pH became ~6) and dried at room temperature. GO paste was then prepared by adding together 0.115 g of ethyl cellulose, 0.6 g of ethanol, 1.1 g of terpineol, and 0.03 g of GO powder. All of the ingredients were mixed and grinded very well in a mortar for 30 min. The final paste was deposited on the carbon paper with a spatula and heated at 340 °C for 30 min. Heating transformed GO into reduced graphene oxide (RGO). The last step was repeated until 0.5 mg of RGO per cm^2^ were deposited. This procedure allowed the construction of a RGO/CP counter electrode.

Another variant was carbon black deposited on a carbon paper electrode (CB/CP): 0.246 g of carbon black was mixed with 8 mL of distilled water by vigorous mixing in a mixer (about 2400 r.p.m.) until it became a viscous paste. This paste was further mixed with 0.088 mL of polytetrafluorethylene (Teflon 60 wt. % dispersion in water), and then applied on a carbon paper cut in the necessary dimensions. This has been achieved by first spreading the paste with a spatula, preheating for a few minutes at 80 °C, and finally heating also for a few minutes in an oven at 340 °C. The total quantity of the deposited CB was again 0.5 mg per cm^2^.

A third variant consisted of the addition of Pt on the CB/CP electrode (Pt/CB/CP): 1 g of Pt–carbon black electrocatalyst (30% on Vulcan XC72) was mixed with 8 g of Nafion perfluorinated resin (5 wt. % solution in lower aliphatic alcohols and water) and 15 g of a solution made of 7.5 g H_2_O and 7.5 g isopropanol. The mixture was ultrasonically homogenized and then applied on carbon black. The electrode was then preheated for a few minutes at 80 °C, finally heated also for a few minutes in an oven at 340 °C, and the procedure was repeated as many times as necessary to load about 0.5 mg of catalyst/cm^2^.

### 2.4. Description of the Reactor

**Figure 1:**
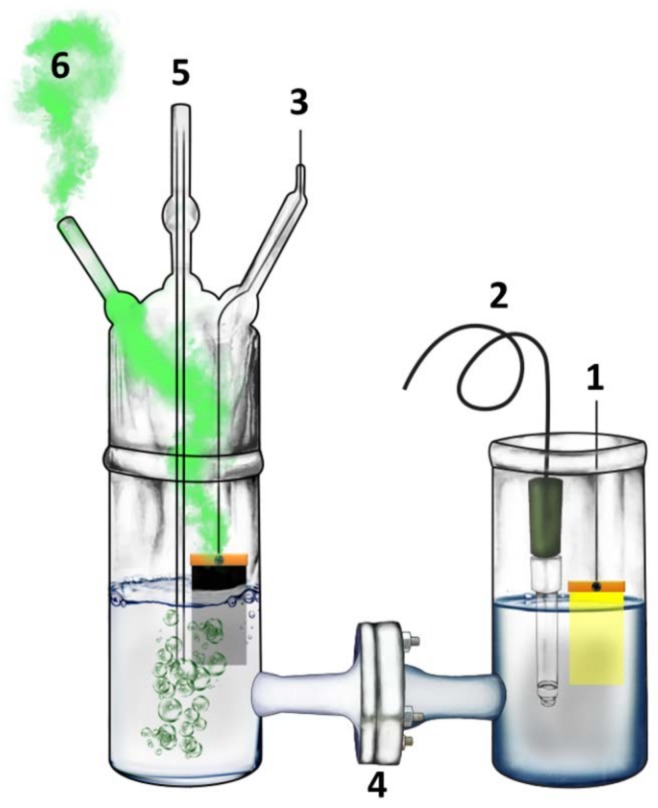
Graphical design of the employed reactor: (1) photoanode; (2) reference electrode; (3) counter electrode; (4) ion transfer membrane; (5) tube for the introduction of Ar; and (6) exhaust of Ar mixed with H_2_.

An H-shaped reactor made of Pyrex glass has been used, as schematically represented by Figure 1. As already said, the electrodes used for hydrogen production had an active area of 3.5 cm × 5 cm, but when current–voltage curves were recorded, the photoanode active area was only 1 cm^2^. Each compartment contained 200 mL of aqueous electrolyte. In most cases, the same alkaline electrolyte was used in both compartments, i.e., 0.5 M NaOH. In the anode compartment, we have also added 10% *v*/*v* ethanol. In some cases, the cathode compartment contained 0.5 M H_2_SO_4_ instead of NaOH in order to apply a chemical bias and provide a favorable environment for hydrogen production by proton reduction. In the case of all of the alkaline electrolytes, the two compartments were separated by a silica frit (ROBU, Hattert, Germany, porosity SGQ 5, diameter 25 mm, thickness 2 mm). When the cathode compartment contained an acidic electrolyte, the ion transfer membrane was a Nafion film (N117, Ion Power, Inc., New Castle, DE, USA). Activation of the Nafion membrane obeyed the following protocol: (1) heating for 1 h at 80 °C in 0.1 M of H_2_O_2_; (2) heating for 1 h at 80 °C in two-dimensional (2D) water; (3) treatment for 1 h at 80 °C in 0.1 M of H_2_SO_4_; and cleaning again for 1 h at 80 °C in 2D water. Thus, the activated Nafion membrane was conserved in 2D water.

Hydrogen was monitored on line by using Ar as a carrier inert gas and applying a bias measured vs. Ag/AgCl. The reference electrode was accommodated in the anode compartment, which was exposed to the ambient, while the cathode compartment was sealed and allowed the flow of Ar using appropriate fittings (Figure 1, cf. Raptis et al. Ref [22]). Illumination was made in all cases using a Xe lamp providing an intensity of 100 mW cm^−2^ at the position of the photoanode.

### 2.5. Measurements and Characterizations

Hydrogen was detected by using an SRI 8610C gas chromatograph. Calibration of the chromatograph signal was accomplished by comparison with a standard of 0.25% H_2_ in Ar. The application of electric bias and current–voltage curves were traced with the help of an Autolab potentiostat PGSTAT128N. Diffuse reflectance absorption spectra were recorded with a Shimadzu UV-2600 spectrophotometer (Shimadzu Corp., Kyoto, Japan) equipped with an integration sphere, and SEM images were obtained with a Zeiss EVO MA-10 microscope (Zeiss, Oberkochen, Germany).

## 3. Results and Discussion

### 3.1. Materials Characterization

**Figure 2:**
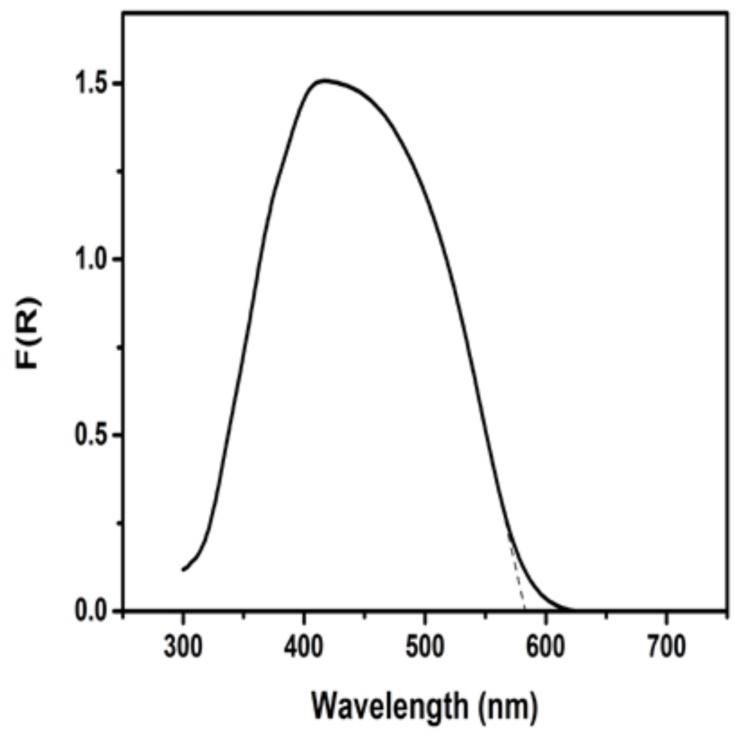
Diffuse reflectance absorption spectrum of the combined CdS/TiO_2_ photocatalyst film.

The nanoparticulate titania film deposited on the photoanode electrode was, as already said, approximately 10-µm thick and presented a mesoporous structure, as typical of films made of P25 nanoparticles. SEM images of such films have already been reported, for example, in Refs [23,24,25]. CdS was formed within the mesoporous structure of titania and presented the spectroscopic characteristics of Figure 2. The combined CdS/TiO_2_ photocatalyst could then absorb photons up to about 580 nm. The maximum photocurrent expected for such a combined semiconductor and for this spectral range can be related to published charts [26] and mounts up to about 12 mA cm^−2^. This current may be even larger because of current doubling phenomena in the presence of a fuel. Current doubling is the name given to an increase of the current due to the injection of additional electrons into the conduction band of the photocatalyst. These electrons derive from unstable radicals that are formed during oxidation of the fuel [19].

**Figure 3:**
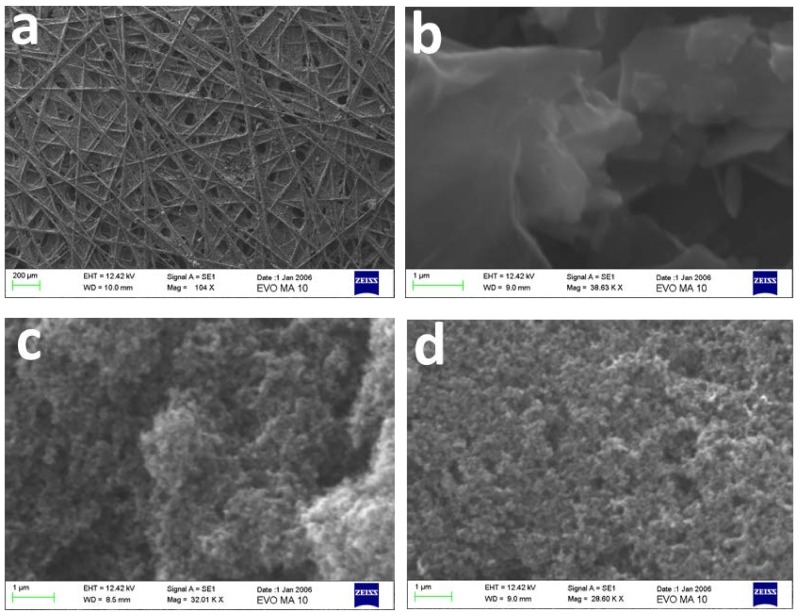
SEM images of the carbon paper used in this work: (**a**) plain and (**b**–**d**) covered with various electrocatalysts: (**b**) reduced graphene oxide (RGO); (**c**) carbon black; and (**d**) carbon black and Pt. The scale bar is 200 µm in (**a**) and 1 µm in (**b**–**d**).

The carbon paper employed as the counter electrode in the present work possesses the structure described by the SEM image of Figure 3a. The addition of electrocatalyst film did not present any unusual features, but demonstrated the typical characteristics expected for the materials used. Thus, graphene sheets are distinguished in Figure 3b, and carbon nanoparticles are distinguished in Figure 3c. The features of Figure 3d are similar to those of Figure 3c, since Pt nanoparticles cannot be imaged by the present SEM technique.

### 3.2. Current–Voltage Characteristics of the Photoelectrochemical Cell

**Figure 4:**
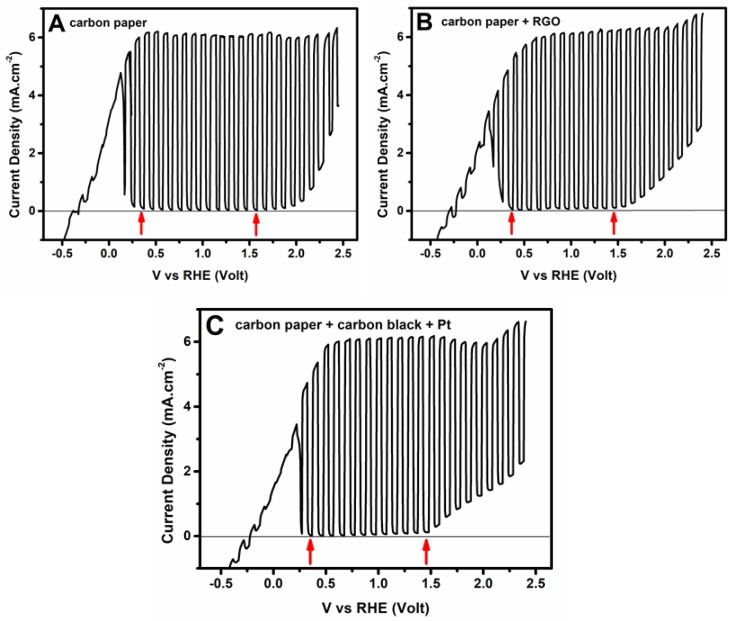
Current density–voltage curves recorded by a light chopping mode with the same CdS/TiO_2_/FTO photoanode, but with different counter electrodes: (**A**) carbon paper; (**B**) carbon paper + RGO; and (**C**) carbon paper + carbon black + Pt. The electrolyte was 0.5 M NaOH. 10% *v*/*v* ethanol was added in the anode compartment. The scan rate was 5 mV/s. In each plot, there are two arrows. The arrow on the left indicates the voltage where the capacitance current becomes zero. The arrow on the right shows the voltage where water electrolysis begins.

Current–voltage curves were recorded in a three-electrode configuration using a small photoanode with an active surface equal to 1 cm^2^. Curves were recorded by light chopping in order to detect the conditions for the production of a photocurrent. Some of the obtained results are presented in Figure 4. Recording was actually made by using a Ag/AgCl reference electrode, but the data were referenced to Reversible Hydrogen Electrode (RHE) by employing the formula

V_RHE_ (Volts) = V_Ag/AgCl_ + 0.2 + 0.059 × (pH) (6)

The pH of the alkaline electrolyte in the present case was 12.0; therefore, 0.91 Volts were added to the values of voltage vs. Ag/AgCl. The vertical arrows in the plots of Figure 4 define the voltage range where a pure photocurrent was produced. Below this range, there is a capacitance current deriving from a reversible intercalation of cations into the mesoporous structure of the photocatalyst film. This is a systematic observation with mesoporous oxide semiconductors, and it is more impressive in the case of titania and WO_3_ [25,27]. At negative voltages, cations are adsorbed into the mesoporous structure, creating an anodic current. The current goes to zero at positive voltage, and the cations are expelled from the film. Cation adsorption is accompanied by a reversible change in the color of the photoanode, i.e., it demonstrates the well-known electrochromic effect [28,29,30,31]. Above the range of the pure photocurrent, water electrolysis was obtained, which progressively prevailed in the observed current. Pure photocurrent was obtained approximately between 0.35–1.5 V vs. RHE. This range was same in all of the cases studied, within experimental error. It is explained by the fact that the electrolyte in the anode compartment was always the same and the reference electrode was placed close to the photoanode.

The anodic photocurrent progressively increased with applied voltage and attained the highest value at about 0.5 V vs. RHE. After that, the current remained stable within the whole range of the pure photocurrent. Therefore, all of the samples gave a stable photocurrent in the range of 0.5 V to 1.5 V vs. RHE. The latter was naturally chosen as the range of preferable electric bias to produce hydrogen. Indeed, hydrogen production was monitored at three applied voltages, i.e., 0.5 V, 1.0 V, and 1.5 V vs. RHE. The current (in reality, current density) recorded by the plots of Figure 4 is the current flowing between the photoanode and the counter electrode. Interestingly, the maximum current was within the experimental error the same for all of the cases of counter electrode studied. At first glance, this is surprising, since the counter electrode, for example, which carried a powerful electrocatalyst such as Pt/carbon black, might facilitate higher current flow. This was presently not the case. We believe that the present system has achieved current saturation, and for this reason, the plot was flat within a large voltage range. Whatever differentiation between different counter electrodes may then demonstrate itself in hydrogen production efficiency.

Before moving to the next section, it is worth commenting on the maximum current density attained by the present reactor, which was approximately 6.3 mA cm^−2^. In the previous section, it was said that maximum current expectancy, according to the spectral absorption range of the combined CdS/TiO_2_ photocatalyst, was 12 mA cm^−2^, and it may even be larger due to the current doubling phenomena. It is, of course, not a surprise that this maximum was presently not reached. The reason is the high rate of electron-hole recombination, which is partially limited by the presence of the fuel, but it was not completely eliminated.

### 3.3. Photoelectrochemical Hydrogen Production

**Figure 5:**
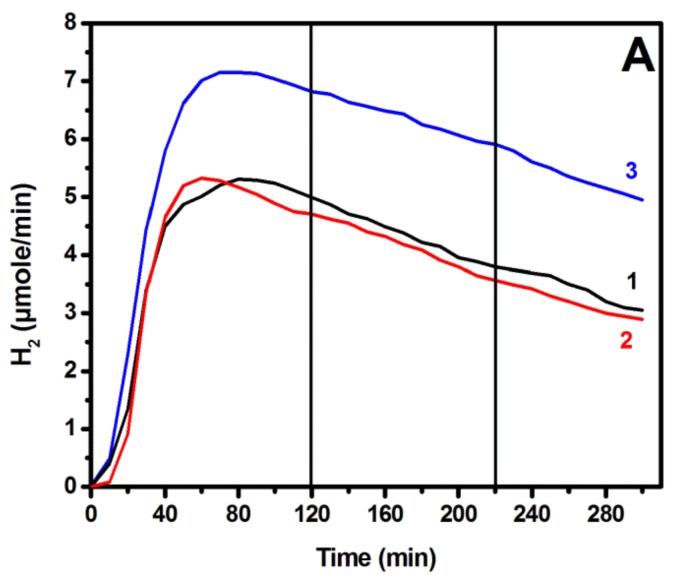

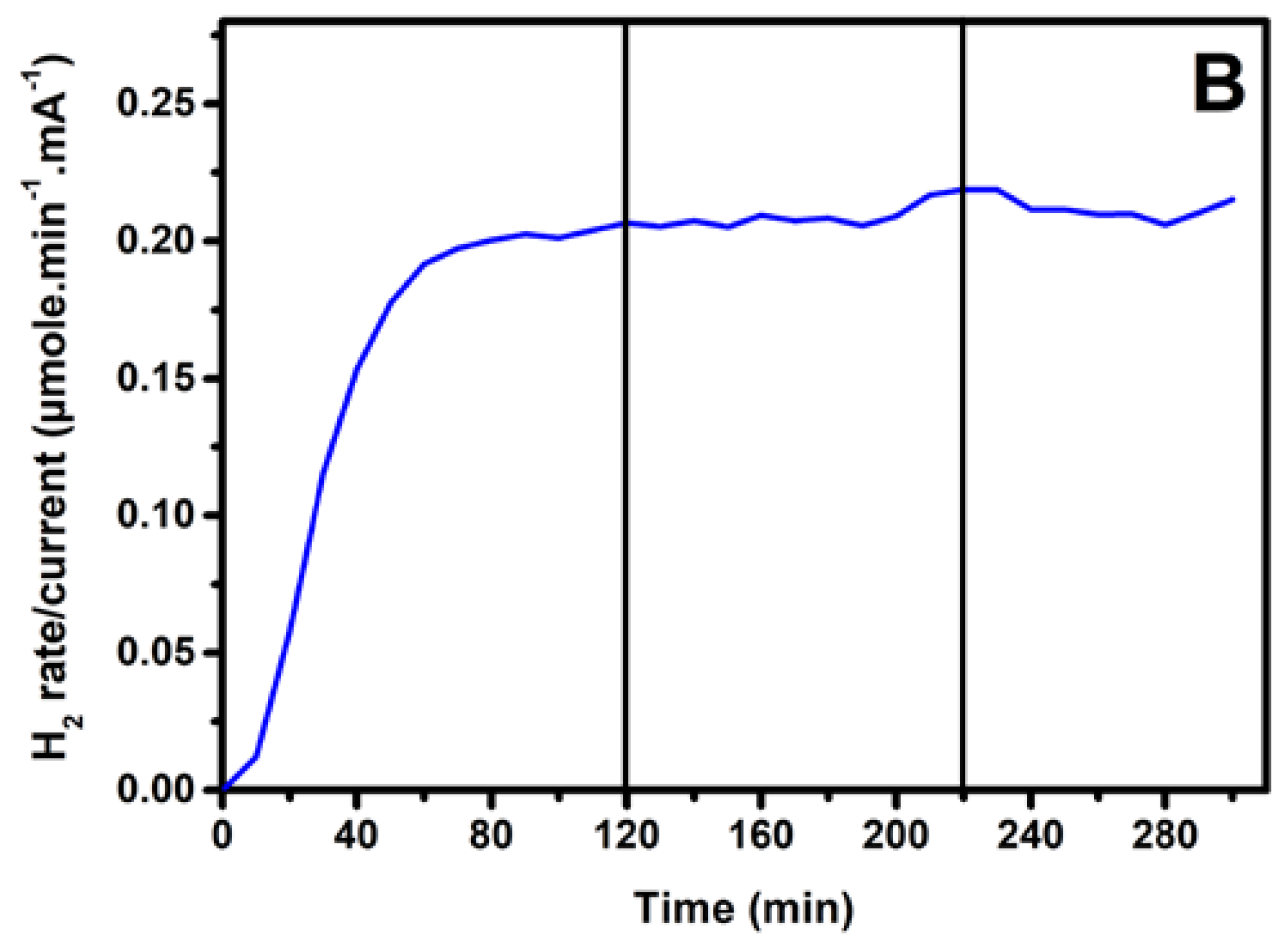
(**A**) Hydrogen production rates recorded by using the same photoanode and three different counter electrodes: (1) carbon paper without electrocatalyst; (2) carbon paper and RGO; and (3) carbon paper and carbon black + Pt. (**B**) Plot of the hydrogen production rate (3) divided by the corresponding current. The vertical lines show the times when the applied bias was changed: 0.5 V→1.0 V→1.5 V vs. RHE.

Hydrogen was monitored on line using the reactor of Figure 1. As already said, anode and counter electrodes had in that case an active area of 3.5 cm × 5 cm, while the same always combined CdS/TiO_2_ photocatalyst was deposited on the anode electrode. Measurements were made as a function of the applied electric bias for several different counter electrodes. For most of the measurements, the same alkaline electrolyte was used in both cell compartments, but in some cases that will be defined below, in the cathode (counter electrode) compartment, an acidic electrolyte was instead used. Figure 5A shows the hydrogen production rate as a function of time for some selected counter electrodes, namely, a carbon paper alone, a carbon paper carrying RGO, and a carbon paper carrying a Pt/carbon black electrocatalyst. The addition of RGO did not affect the performance of carbon paper alone, but the presence of Pt/carbon black did result in a higher hydrogen production rate, as expected. The addition of carbon black alone, without Pt (not shown), did not much affect the hydrogen production rate either. Therefore, it was Pt that made the difference while carbonaceous nanoparticulate films did not add to the inherent capacity of carbon paper alone to act as a functional counter electrode. Each curve of Figure 5A consists of a rising part, which corresponds to the accumulation of hydrogen in the device tubes, a maximum, and a slowly dropping part. The decrease of the hydrogen production rate was approximately proportional to the decrease of the current flowing between the working and the counter electrode. Thus, by dividing the recorded hydrogen rate by the corresponding current, we obtained the curve of Figure 5B. After achieving the maximum, this curve remained practically stable, thus verifying the proportionality between current and hydrogen rate. This result reveals that the main reason for the decrease of the hydrogen production rate is the decrease of the current. Hydrogen is produced by reduction reactions taking place at the counter electrode, assisted by the arriving electrons. Thus, it is not a surprise that the variation of hydrogen production rate goes in parallel with the variation of the current. The arising question is then: what causes the current decrease? One main reason is the consumption of the fuel (ethanol). This can be shown by adding fresh electrolyte with 10% v/v ethanol. The current then (not shown) underwent a 20% increase. However, degradation of the photocatalyst is another main reason for current drop, and this was again demonstrated by introducing a freshly prepared photoanode. Indeed, with a fresh photoanode and a fresh electrolyte, the original photocurrent was recovered.

In the plots of Figure 5, there are two vertical lines at 120 min and 220 min. These lines mark the times when the applied bias was changed from 0.5 V to 1.0 V, and then to 1.5 V vs. RHE. It is very interesting that in spite of this drastic modification of the applied voltage, no effect was produced on the current or the hydrogen production rate. The evolution of both continued as if no change to the bias were made. This result is, of course, explained by the flatness of the plots of Figure 4 within the range 0.5 V to 1.5 V vs. RHE. The current was then expected to remain the same by increasing the bias within that range, and it did. The hydrogen production rate followed suite. The consistency then between the data of Figure 4 and Figure 5 comes in support of the above hydrogen production model. Hydrogen can obviously be produced equally well by applying the lowest possible electric bias of only 0.5 V vs. RHE.

Photoelectrochemical hydrogen production relates hydrogen production rate to the flowing electric current. In the ideal case, the formation of one H_2_ molecule corresponds to a current of two electrons, according to reactions (4) or (5). A formula of practical use may then be introduced by the following equivalent: *1 µmole/min H_2_ corresponds to 2 × 10^−6^ × 6.022 × 10^23^ × 1.6 × 10^−19^ C/60 s, which is equal to 3.21 mA*. The actual recorded current was in fact higher than the above relation shows. The Faradaic efficiency for hydrogen production in each recorded case is the ratio of the corresponding ideal current over the actual measured current. The Faradaic efficiencies for photoelectrochemical hydrogen production were calculated in all of the recorded cases, and make the data of Table 1. It is then verified that the most efficient hydrogen production was obtained in the presence of a platinum catalyst. Nevertheless, for a biased hydrogen production, it suffices to use a simple carbon paper to achieve substantial production rates, which were only 24% lower than in the presence of Pt. On the other hand, the addition of carbonaceous materials as electrocatalysts on carbon paper did not offer much advantage. The introduction of an acidic electrolyte into the counter electrode compartment did not produce any impressive effects, even though the plethora of protons in that case might facilitate molecular hydrogen production. The presence of acid did have some favorable effect in the case of plain carbon paper, but it had an adverse effect in the presence of platinum. It is concluded that the alkaline electrolyte did not hinder hydrogen production rates, at least under the present conditions.

## 4. Conclusions

In the present work, we have shown that hydrogen can be photoelectrochemically produced by using a simple carbon paper as counter electrode. A platinum electrocatalyst is more effective, but plain carbon paper is only 24% less effective; therefore, carbon paper is very interesting for practical applications thanks to its low cost. Hydrogen is generated under electric bias. Current flow and hydrogen production rate was flat within a range of 0.5 V to 1.5 V vs. RHE; therefore, it suffices to apply the lowest voltage of this range to obtain a maximum hydrogen production rate. The Faradaic efficiency was practically not affected by the pH value at which hydrogen was produced. Therefore, the reactor can be simplified by applying the same electrolyte both at the working and the counter electrode compartment. An alkaline electrolyte is preferable in that case, since photocatalytic oxidation of the fuel is favored at high hydroxyl ion concentration. An important conclusion to be drawn by the present data is that the differentiation between electrodes and electrocatalysts demonstrates itself mainly by the Faradaic efficiency. The biased current itself may reach saturation in a given device, and thus become less sensitive to the quality of the electrocatalyst.

## Figures and Tables

**Table 1:** Faradaic efficiency for photoelectrochemical hydrogen production by reduction reactions at the counter electrode at various pH values and with various counter electrodes.

Type of Counter Electrode	Type of Electrolyte in the Counter Electrode Compartment	Faradaic Efficiency of Hydrogen Production
Carbon paper	NaOH	51%
Carbon paper + RGO	NaOH	52%
Carbon paper + Carbon Black	NaOH	53%
Carbon paper + Carbon Black + Pt	NaOH	67%
Carbon paper	H_2_SO_4_	57%
Carbon paper + Carbon Black + Pt	H_2_SO_4_	60%
